# Metabolic characteristics and factors associated with prediabetes in Chinese adults based on real-world health examination data: a cross-sectional study

**DOI:** 10.3389/fnut.2026.1850839

**Published:** 2026-06-12

**Authors:** Xinru Sun, Jun Zhou, Zhaoyi Chen, Zeyin Xin, Jiayi Ren, Yuhong Zheng, Jinkun Wang, Jiarun Zhang, Yuxin Zhang, Lu Liu, Bin Li, Yizhan Wang

**Affiliations:** 1 Center of Acupuncture and Moxibustion, Beijing Hospital of Traditional Chinese Medicine, Capital Medical University, Beijing, China; 2 Beijing Key Laboratory of Digital and Intelligent Acupuncture, Beijing, China; 3 Graduate School, Beijing University of Chinese Medicine, Beijing, China; 4 Preventive Treatment Center, Beijing Hospital of Traditional Chinese Medicine, Capital Medical University, Beijing, China

**Keywords:** associated factors, machine learning, metabolic characteristics, prediabetes, regression analysis

## Abstract

**Background:**

Prediabetes is a common intermediate state of abnormal glucose metabolism, but its metabolic characteristics and associated factors in health examination populations remain insufficiently defined. Based on a health examination population of Chinese adults, this study aimed to characterize the metabolic profile of prediabetes and identify factors associated with prediabetes using real-world health examination data, thereby providing a foundation for future multicenter, multiregional, and broader population-based validation and implementation studies.

**Methods:**

This cross-sectional study included adults undergoing routine health examinations between January 1, 2018 and December 31, 2024. Metabolic characteristics were compared between normoglycemic individuals and those with prediabetes. Univariable and multivariable logistic regression analyses were performed to identify factors associated with prediabetes. XGBoost combined with SHAP was further used to assess the relative importance of clinical and metabolic indicators.

**Results:**

A total of 20,271 participants were included in the main analysis, including 8,457 with prediabetes and 11,814 with normoglycemia. Compared with the normoglycemia group, the prediabetes group was older (58.48 ± 13.10 vs. 48.10 ± 12.90, *P* < 0.001), had a higher BMI (26.82 ± 3.78 vs. 22.83 ± 3.95, *P* < 0.001) and was more likely to have hypertension and fatty liver. The prediabetes group also had higher TC, TG, and LDL-C levels and lower HDL-C and TBIL levels (*P* < 0.001). In multivariable analyses, BMI (aOR 2.52, 95% CI 2.42–2.61), fatty liver (aOR 2.80, 95% CI 2.60–3.02), TC (aOR 1.08, 95% CI 1.05–1.12), TG (aOR 1.09, 95% CI 1.05–1.12), and LDL-C (aOR 1.17, 95% CI 1.13–1.21), age, hypertension, ALT, and BUN, were positively associated with prediabetes, whereas HDL-C (aOR 0.84, 95% CI 0.81–0.87) and TBIL (aOR 0.75, 95% CI 0.72–0.78) were inversely associated. Among the extended models, the model additionally including BMI showed the best discriminative performance, with the AUC increasing from 0.763 to 0.825. The XGBoost model achieved an AUC of 0.941 (95% CI 0.934–0.948), and BMI, age, TG, fatty liver, and TC ranked among the most informative variables. SHAP dependence analysis showed that the contribution of BMI to model output was nonlinear, becoming positive at approximately 23.9 kg/m^2^, peaking near 29.4 kg/m^2^, and attenuating thereafter, whereas age showed a generally monotonic positive relationship with model output and crossed zero at approximately 54 years.

**Conclusion:**

In this health examination population, prediabetes was associated with age, hypertension, BMI, fatty liver, lipid profiles, and altered hepatorenal function-related indicators. Among these factors, BMI and fatty liver showed relatively stronger associations, and machine-learning analysis further highlighted BMI, age, TG, fatty liver, and TC as particularly informative variables for the early identification of prediabetes.

## 1 Introduction

Prediabetes is an intermediate state of abnormal glucose metabolism between normal glucose homeostasis and overt diabetes mellitus (1). According to the latest global estimates, the prevalence of impaired glucose tolerance (IGT) and impaired fasting glucose (IFG) was 12.0% and 9.2%, respectively, and both are projected to continue increasing through 2050 (2). Accumulating evidence indicates that prediabetes increases the risk of cardiovascular disease (CVD), chronic kidney disease (CKD), as well as early mortality (3, 4).

Prediabetes is widely regarded as a critical and potentially reversible stage in the progression to type 2 diabetes mellitus (T2DM). In a recent pooled analysis of individual-level data from 19 prospective cohort studies, the 10-year probability of progression from prediabetes to T2DM was 12.5%, whereas the probability of reversion to normoglycemia was 36.1%. These findings underscore the reversible nature of prediabetes and highlight an important opportunity for early identification and preventive intervention to delay or prevent the onset of diabetes (5). The development of prediabetes cannot be attributed to abnormal glucose metabolism alone, but rather reflects the combined effects of genetic susceptibility, aging, obesity, dyslipidemia, hypertension, fatty liver disease, and indicators related to hepatic and renal function (6, 7). Characterizing these metabolic characteristics may help improve early risk assessment and support timely intervention. In addition to conventional anthropometric and lipid measurements, biochemical markers routinely obtained during health examinations may provide complementary information on early metabolic abnormalities. Elevated serum uric acid levels have been strongly correlated with an accelerated progression from prediabetes to overt diabetes (8). Altered liver enzyme profiles are established predictors of incident type 2 diabetes across diverse populations (9). Moreover, evidence from health examination cohorts suggests that composite metabolic indices may help identify individuals at elevated risk of prediabetes and diabetes in real-world settings (10–12).

Despite previous research on individual biomarkers and surrogate indices, a comprehensive understanding of the metabolic characteristics and associated factors of prediabetes based on real-world health examination data remains limited. Therefore, the present study aimed to characterize the metabolic characteristics of adults with prediabetes and to identify factors associated with prediabetes based on real-world health examination data from a large cross-sectional study population.

## 2 Materials and methods

### 2.1 Study design

This single-center retrospective cross-sectional study was conducted at Beijing Hospital of Traditional Chinese Medicine, Capital Medical University, and was jointly performed by the Center of Acupuncture and Moxibustion and Preventive Treatment Center. The results are reported in accordance with the STROBE (Strengthening the Reporting of Observational Studies in Epidemiology) statement for cross-sectional studies (https://www.strobe-statement.org/), and the checklist is provided in Supplementary Table S1.

### 2.2 Data sources and measurements

This study collected relevant data from the health examination population at Beijing Traditional Chinese Medicine Hospital affiliated to Capital Medical University from January 1, 2018 to December 31, 2024. The data mainly included demographic parameters, lifestyle-related information, physical examination metrics, abdominal ultrasound findings, and laboratory test indicators, involving glucose levels, lipid profiles, and hepatic and renal function assessments. All measurements were performed using the same procedures for all participants. Prediabetes was defined based on both the American Diabetes Association (ADA) guidelines (1) and the World Health Organization (WHO) criteria (13). It was identified using available glycemic measurements, namely a hemoglobin A1c (HbA1c) level of 5.7–6.4% and/or a fasting plasma glucose (FPG) level of 100–125 mg/dL (5.6–6.9 mmol/L). Participants who met the diagnostic criteria for prediabetes were classified into the Prediabetes Group, whereas those with normal glycemia were classified into the Normoglycemia Group.

### 2.3 Participants

Health examination participants were included if they met the following criteria: (1) age ≥18 years; (2) complete data on FPG and HbA1c for glycemic classification; (3) complete data on key covariates including total cholesterol (TC), triglycerides (TG), high-density lipoprotein cholesterol (HDL-C), low-density lipoprotein cholesterol (LDL-C), body mass index (BMI), fatty liver status, and basic demographic characteristics.

The exclusion criteria were as follows: (1) participants with a prior diagnosis of diabetes mellitus (DM); (2) those whose HbA1c level at the health examination met the diagnostic threshold for DM (≥6.5%), and those whose FPG level met the diagnostic threshold for DM (≥126 mg/dl [7.0 mmol/L]); (3) women who were pregnant or lactating; (4) a documented diagnosis of severe cognitive impairment in the medical records.

### 2.4 Sample size

Prior research suggests that the prevalence of prediabetes in China is estimated to be 38.1% (14). Based on this estimate, the sample size was calculated using the formula for cross-sectional studies based on prevalence. Assuming an expected prevalence of prediabetes of *p* = 38.1% (0.381), a significance level of α =0.05, Z_1−α/2_ = 1.96 and an allowable error of d = 10% × *p* = 0.0381, the minimum required sample size was calculated as *n* = 625. After accounting for a 10% invalid data rate, at least 694 participants were required for inclusion. Although the minimum required sample size was achieved, the larger sample is expected to improve estimate precision and further enhance the robustness of the findings.

### 2.5 Statistical methods

Prediabetes was treated as a binary outcome variable, with normoglycemic individuals serving as the reference group. Continuous variables were summarized as mean ± standard deviation (SD) or median (interquartile range [IQR]) according to their distributional characteristics, whereas categorical variables were presented as counts and percentages. For between-group comparisons, normally distributed continuous variables were analyzed using the independent-samples *t* test, non-normally distributed continuous variables were compared using the Wilcoxon rank-sum test, and categorical variables were compared using Pearson's χ^2^ test.

To investigate factors linked to prediabetes, univariable binary logistic regression was initially employed to evaluate the relationships between each indicator and prediabetes, subsequently followed by multivariable binary logistic regression analysis. A base model was subsequently developed by incorporating age, gender, hypertension, and indicators linked to hepatorenal function, including TBIL, AST, ALT, BUN, SCR, and UA. Utilizing this model, TC, TG, LDL-C, HDL-C, fatty liver, and BMI were incorporated individually to develop extended models for assessing the relationships between lipid metabolism-related variables and prediabetes. To enhance the comparability of regression coefficients among continuous variables, all continuous variables used in the regression models were standardized by Z-score transformation prior to model fitting. Furthermore, as an ancillary investigation, machine learning methodologies were utilized to investigate the relative significance of clinical and metabolic indicators. An extreme gradient boosting (XGBoost) model was constructed, and SHapley Additive exPlanations (SHAP) were employed for model interpretation to demonstrate the relative contributions and directional influences of significant lipid and metabolic indicators on the projected likelihood of prediabetes. All statistical analyses were conducted utilizing R software (version 4.3.0).

## 3 Results

### 3.1 General information

**Figure 1:**
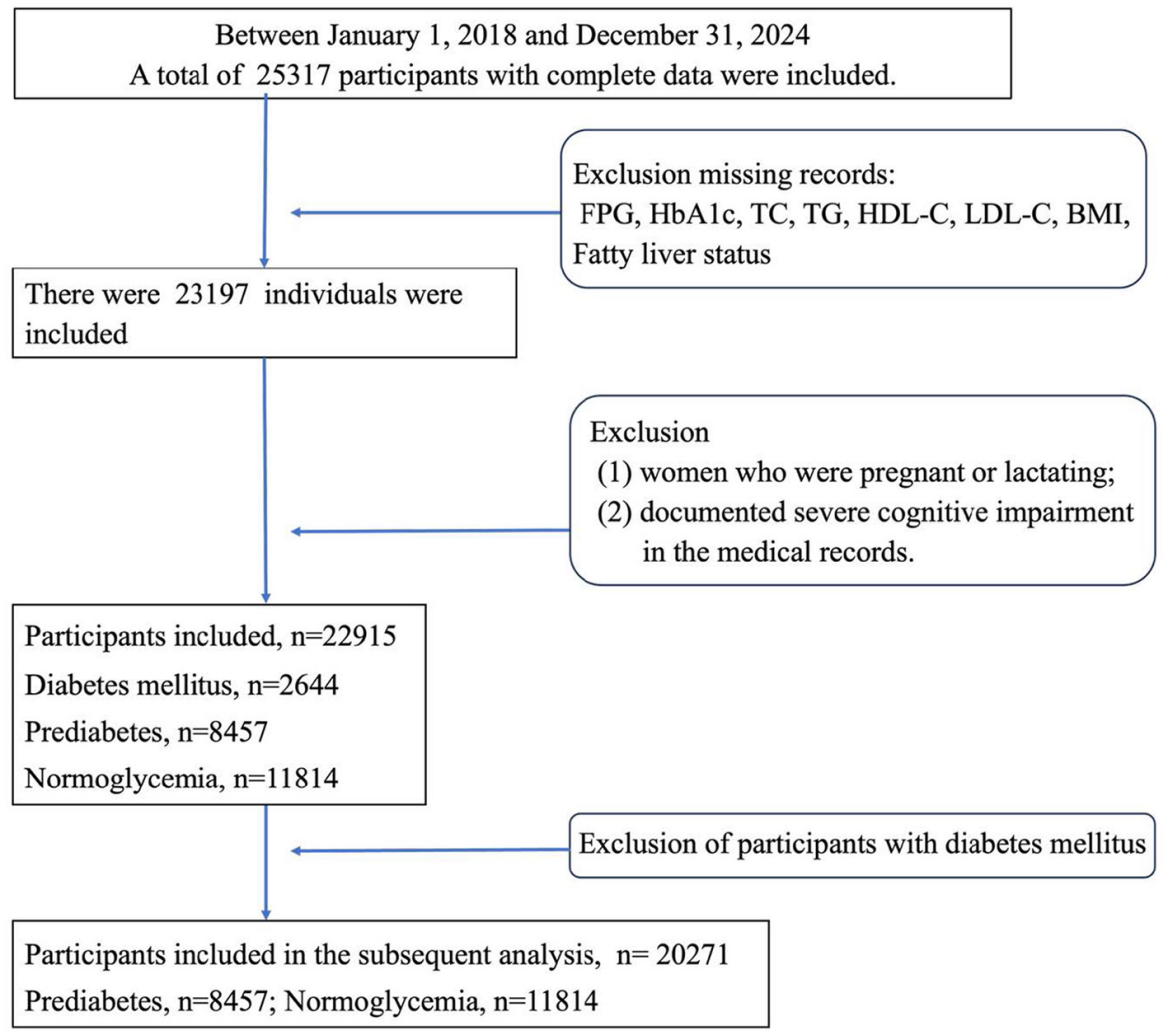
The study flow chart.

A total of 25,317 participants were initially included. After excluding 1,422 participants with missing FPG and/or HbA1c data, 698 with missing key diabetes-related variables, including lipid profile parameters and BMI, and 282 who were pregnant or lactating, or had cognitive or communication impairments, 22,915 participants were available. Among these participants, 8,457 as having prediabetes. The prevalence of prediabetes in the eligible study population was therefore 36.9% (8,457/22,915). For the subsequent analyses, 2,644 participants classified as having diabetes were excluded, leaving 20,271 participants in the final analytical sample, including 8,457 with prediabetes and 11,814 with normoglycemia. Details are shown in Figure 1.

### 3.2 Baseline characteristics of participants

The prediabetes group was markedly older than the normoglycemia group (58.48 ± 13.10 vs. 48.10 ± 12.90 years, *P* < 0.001) and had a slightly higher proportion of men (42.6% vs. 40.3%, *P* = 0.001). The prediabetes group had a higher BMI than the normoglycemia group (26.82 ± 3.78 vs. 22.83 ± 3.95 kg/m^2^, *P* < 0.001). No significant differences were observed between the two groups in smoking history or alcohol drinking (*P* > 0.05). The proportions of participants with hypertension (57.6% vs. 36.9%, *P* < 0.001) and fatty liver (38.2% vs. 15.4%, *P* < 0.001) were both significantly higher in the prediabetes group than in the normoglycemia group. The prediabetes group had lower TBIL levels [14.60 (11.90, 18.20) vs. 15.70 (12.60, 19.40), *P* < 0.001] and HDL-C levels [1.38 (1.18, 1.63) vs. 1.45 (1.23, 1.71), *P* < 0.001], whereas ALT, AST, BUN, Scr, UA, TC [5.14 (4.44, 5.82) vs. 4.81 (4.23, 5.45), *P* < 0.001], TG [1.42 (1.03, 1.99) vs. 1.09 (0.78, 1.58), *P* < 0.001], and LDL-C levels [3.03 (2.49, 3.59) vs. 2.73 (2.27, 3.24), *P* < 0.001] were significantly higher in the prediabetes group (all *P* < 0.001). Details are shown in Table 1.

**Table 1:** Baseline characteristics of participants by group.

Variables	Total (*n* = 20,271)	Prediabetes group (*n* = 8,457)	Normoglycemia group (*n* = 11,814)	*P* value
Age (years)	52.00 (41.00, 63.00)	58.48 ± 13.10	48.10 ± 12.90	<0.001
Gender				
Female	11,900 (58.7%)	4,851 (57.4%)	7,049 (59.7%)	0.001
Male	8,371 (41.3%)	3,606 (42.6%)	4,765 (40.3%)	
BMI (kg/m2)	23.67 (21.38, 28.40)	26.82 ± 3.78	22.83 ± 3.95	<0.001
Smoking history				
No	14,265 (70.4%)	5,910 (69.9%)	8,355 (70.7%)	0.197
Yes	6,006 (29.6%)	2,547 (30.1%)	3,459 (29.3%)	
Alcohol drinking				
No	12,032 (59.4%)	4973 (58.8%)	7,059 (59.8%)	0.176
Yes	8,239 (40.6%)	3484 (41.2%)	4,755 (40.2%)	
Hypertension				
No	11,040 (54.5%)	3582 (42.4%)	7,458 (63.1%)	<0.001
Yes	9,231 (45.5%)	4875 (57.6%)	4,356 (36.9%)	
Fatty liver				
No	15,217 (75.1%)	5,228 (61.8%)	9,989 (84.6%)	<0.001
Yes	5,054 (24.9%)	3,229 (38.2%)	1,825 (15.4%)	
TBIL (μmol/L)	15.20 (12.30, 19.00)	14.60 (11.90, 18.20)	15.70 (12.60, 19.40)	<0.001
ALT (U/L)	17.80 (13.00, 25.90)	19.80 (14.50, 28.60)	16.40 (12.10, 23.90)	<0.001
AST (U/L)	20.40 (17.40, 24.70)	21.50 (18.30, 26.10)	19.70 (16.80, 23.67)	<0.001
BUN (mmol/L)	4.75 (4.01, 5.58)	4.95 (4.21, 5.82)	4.60 (3.88, 5.40)	<0.001
Scr (μmol/L)	63.00 (54.20, 74.00)	64.00 (55.00, 74.40)	62.00 (53.90, 73.30)	<0.001
UA (μmol/L)	320.90 (266.00, 390.00)	333.00 (277.40, 401.00)	311.00 (257.90, 382.00)	<0.001
TC (mmol/L)	4.93 (4.31, 5.62)	5.14 (4.44, 5.82)	4.81 (4.23, 5.45)	<0.001
TG (mmol/L)	1.22 (0.86, 1.76)	1.42 (1.03, 1.99)	1.09 (0.78, 1.58)	<0.001
HDL-C (mmol/L)	1.42 (1.21, 1.68)	1.38 (1.18, 1.63)	1.45 (1.23, 1.71)	<0.001
LDL-C (mmol/L)	2.85 (2.34, 3.40)	3.03 (2.49, 3.59)	2.73 (2.27, 3.24)	<0.001

Comparison of baseline clinical and metabolic characteristics between the normoglycemia group and prediabetes group.

BMI, body mass index; TBIL, total bilirubin; ALT, alanine aminotransferase; AST, aspartate aminotransferase; BUN, blood urea nitrogen; Scr, serum creatinine; UA, uric acid; TC, total cholesterol; TG, triglycerides; HDL-C, high-density lipoprotein cholesterol; LDL-C, low-density lipoprotein cholesterol.

### 3.3 Logistic regression analysis of factors associated with prediabetes

**Figure 2:**
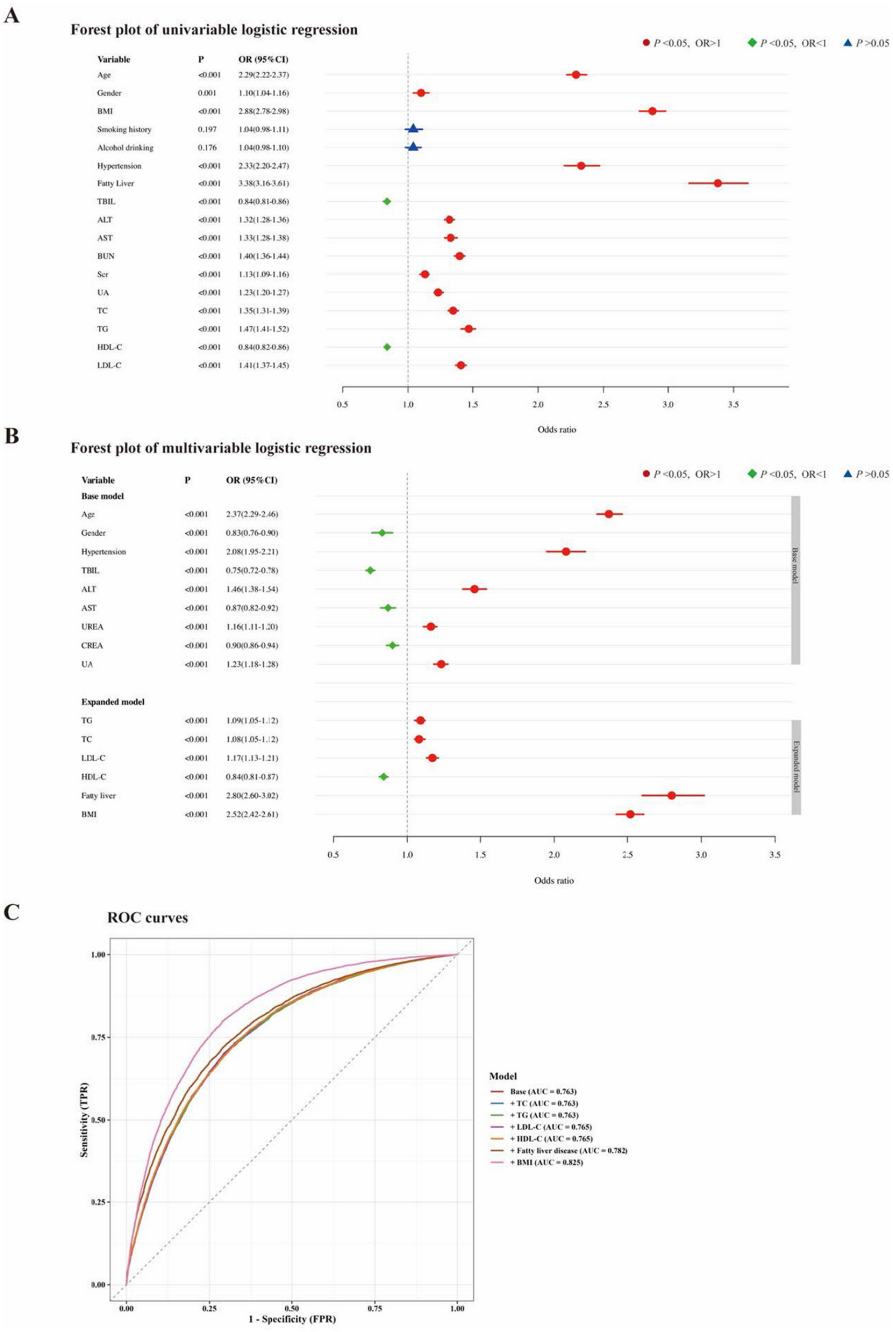
Logistic regression analysis. **(A)** Forest plot of univariable logistic regression analysis. **(B)** Forest plot of multivariable logistic regression analysis. **(C)** The ROC curves of different models. OR, odds ratio; CI, confidence interval.

All variables were first evaluated using univariable logistic regression analysis. age (OR 2.29, 95% CI 2.22–2.37; *P* < 0.001), gender (OR 1.10, 95% CI 1.04–1.16; *P* = 0.001), BMI (OR 2.88, 95% CI 2.78–2.98; *P* < 0.001), hypertension (OR 2.33, 95% CI 2.20–2.47; *P* < 0.001), fatty liver (OR 3.38, 95% CI 3.16–3.61; *P* < 0.001), ALT, AST, BUN, Scr, UA, TC (OR 1.35, 95% CI 1.31–1.39; *P* < 0.001), TG (OR 1.47, 95% CI 1.41–1.52; *P* < 0.001), and LDL-C (OR 1.41, 95% CI 1.37–1.45; *P* < 0.001) were significantly positively associated with prediabetes. Among these variables, fatty liver, BMI, age, and hypertension showed relatively stronger positive associations, with ORs > 2. By contrast, TBIL (OR 0.84, 95% CI 0.81–0.86; *P* < 0.001) and HDL-C (OR 0.84, 95% CI 0.82–0.86; *P* < 0.001) were inversely associated with prediabetes. Smoking history and alcohol drinking were not significantly associated with prediabetes. The univariable logistic regression results are presented in Figure 2A. Variable coding for logistic regression analysis is provided in Supplementary Table S2, and the detailed results are presented in Supplementary Table S3.

In the multivariable logistic regression analysis, the base model showed that age (aOR 2.37, 95% CI 2.29–2.46; *P* < 0.001), hypertension (aOR 2.08, 95% CI 1.95–2.21; *P* < 0.001), ALT (aOR 1.46, 95% CI 1.38–1.54; *P* < 0.001), BUN (aOR 1.16, 95% CI 1.11–1.20; *P* < 0.001), and UA (aOR 1.23, 95% CI 1.18–1.28; *P* < 0.001) remained positively associated with prediabetes. Among these variables, age and hypertension showed relatively stronger positive associations, with aORs > 2. When BMI, fatty liver, TC, TG, HDL-C, and LDL-C were individually added to the base model, BMI (aOR 2.52, 95% CI 2.42–2.61; *P* < 0.001), fatty liver (aOR 2.80, 95% CI 2.60–3.02; *P* < 0.001), TC (aOR 1.08, 95% CI 1.05–1.12; *P* < 0.001), TG (aOR 1.09, 95% CI 1.05–1.12; *P* < 0.001), and LDL-C (aOR 1.17, 95% CI 1.13–1.21; *P* < 0.001) were also positively associated with prediabetes, among which BMI and fatty liver showed relatively stronger positive associations, with aORs > 2. In contrast, gender, TBIL (aOR 0.75, 95% CI 0.72–0.78; *P* < 0.001), AST, and Scr remained inversely associated with prediabetes in the base model, while higher HDL-C (aOR 0.84, 95% CI 0.81–0.87; *P* < 0.001) was inversely associated with prediabetes in the extended model. The multivariable logistic regression results are presented in Figure 2B. Detailed results are shown in Supplementary Table S4.

Among these extended models, the model additionally including BMI showed the best discriminative performance, with the AUC increasing from 0.763 to 0.825, which was higher than that of the models additionally including TC, TG, LDL-C, HDL-C, or fatty liver (Figure 2C).

### 3.4 Machine learning analysis

XGBoost and SHAP analyses showed that the model achieved excellent discrimination in the test set, with an area under the receiver operating characteristic curve (AUC) of 0.941 (95% CI: 0.934–0.948). The optimal cutoff value determined by the Youden index was 0.432. At this threshold, the accuracy, sensitivity, specificity, precision, and F1 score were 0.867, 0.863, 0.870, 0.826, and 0.844, respectively. The corresponding confusion matrix is provided in Supplementary Figure S1.

**Figure 3:**
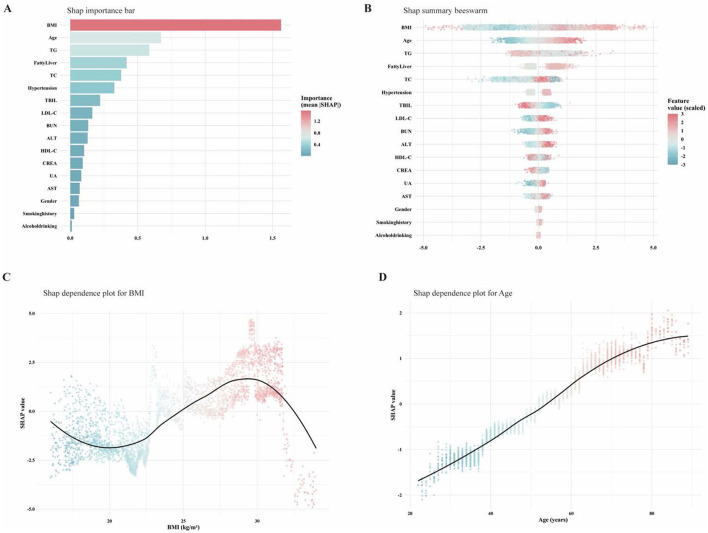
SHAP interpretability analysis of the XGBoost model. **(A)** Feature importance ranking based on mean |SHAP| values. **(B)** SHAP beeswarm summary plot showing the distribution of feature impacts on model predictions. **(C)** SHAP dependence plot for BMI, revealing a non-linear relationship with the model output. **(D)** SHAP dependence plot for Age, illustrating a monotonic increasing trend.

Based on the SHAP analysis, the contributions of individual features to the model output differed substantially. Feature importance ranked by mean absolute SHAP values identified BMI as the most influential variable, followed by age, TG, fatty liver, TC, and hypertension, with TBIL, LDL-C, BUN, ALT, HDL-C, SCR, UA, and AST showing more moderate contributions; gender, smoking history, and alcohol drinking contributed relatively little overall (Figure 3A). Consistently, the SHAP summary plot showed that higher BMI, age, TG, and TC values, as well as the presence of fatty liver and hypertension, were generally associated with higher positive SHAP values, whereas higher TBIL and HDL-C values were generally associated with negative SHAP values (Figure 3B).

SHAP dependence analysis for BMI (Figure 3C) revealed a nonlinear relationship between BMI and the XGBoost model output. The LOESS curve for SHAP(BMI) crossed from negative to positive at approximately 23.9 kg/m^2^, peaked near 29.4 kg/m^2^, declined while remaining predominantly positive, and crossed zero again near 32.3 kg/m^2^. In the post-peak BMI subrange 29.4–32 kg/m^2^ (Supplementary Figure S2), where the marginal contribution of BMI weakened from bin to bin, the mean percentage shares of |SHAP| for TG, TC, and hypertension rose across bins, with hypertension plateauing in the highest bin. By contrast, fatty liver reached its highest share in the 30–31 kg/m^2^ bin and edged down in the 31–32 kg/m^2^ bin, whereas age increased modestly and then plateaued. In contrast, age showed a monotonic increasing relationship with the model output, with SHAP values gradually increasing as age increased, with SHAP values increasing progressively with age and crossing zero at approximately 54 years (Figure 3D).

## 4 Discussion

In this cross-sectional study based on real-world health examination data, prediabetes was associated with age, hypertension, BMI, fatty liver, lipid abnormalities, and several hepatorenal function-related indicators. Taken together, these findings suggest that prediabetes is not merely an abnormality of glycemic regulation, but may also reflect broader metabolic dysregulation. Altered lipid profiles, insulin resistance, visceral and hepatic fat accumulation, and subsequent stress responses in downstream target organs may constitute key pathophysiological features underlying this condition (15).

Among these metabolic changes, BMI emerged as one of the most prominent correlates of prediabetes. BMI was positively associated with prediabetes, showed a relatively large effect size among the extended variables, and ranked among the top predictors in both the XGBoost feature importance and SHAP analyses. The SHAP dependence analysis suggested that the contribution of BMI to model output shifted from negative to positive at approximately 23.9 kg/m^2^. Although this value should not be interpreted as a formal clinical threshold, it may indicate a transition toward a less favorable metabolic profile. This observation is consistent with previous studies showing that overweight status is associated with an increased risk of diabetes and prediabetes (16). Further SHAP dependence analysis indicated that the association between BMI and prediabetes was nonlinear. The attenuation of BMI's marginal contribution after its SHAP peak suggests that, when BMI increases beyond a certain range, BMI alone may provide limited additional information for characterizing metabolic heterogeneity in the model. In the higher BMI range, the relative SHAP contributions of metabolic and clinical features, including TG, TC, hypertension, and fatty liver, became more prominent than that of BMI itself. These patterns imply that, at higher BMI levels, the BMI–prediabetes association may be more closely characterized by concomitant dyslipidemia, blood pressure abnormalities, hepatic steatosis, and age-related metabolic vulnerability than by increased body weight alone. Current evidence suggests that prediabetes often occurs alongside insulin-resistant phenotypes marked by visceral adiposity and ectopic fat accumulation, while obesity-related adipose dysfunction fosters lipolysis, chronic inflammation, adipokine imbalance, and compromised insulin action in various organs (17, 18). Reduction in body weight and central adiposity can improve glycemic status in people at high metabolic risk (19, 20). Therefore, these findings highlight the importance of integrating weight management with comprehensive metabolic assessment and early intervention in health examination populations.

The lipid characteristics associated with prediabetes further suggest that prediabetes is not merely an isolated abnormality of glycemic regulation, but rather an early manifestation of dysregulation of glucose and lipid metabolism. In this study, elevated TC, TG, and LDL-C were positively associated with prediabetes, whereas HDL-C was inversely associated, indicating that adverse lipid remodeling may already be present at the prediabetic stage. Previous studies have likewise shown that individuals with prediabetes may already exhibit a more atherogenic lipid profile, characterized by higher TG, TC, and LDL-C levels together with lower HDL-C levels (21), while even individuals at an earlier dysglycemic stage, such as those with normal glucose tolerance but elevated 1-h post-load glucose, may show lipid patterns resembling those of prediabetes (22). In addition, prospective studies in prediabetic populations have shown that higher TG/HDL-C-related indices are associated with a greater risk of progression to diabetes (23, 24), further supporting the metabolic relevance of elevated TG and reduced HDL-C during the prediabetic stage. Elevated TG may reflect impaired lipid handling and insulin resistance, whereas reduced HDL-C may indicate diminished reverse cholesterol transport and poorer overall metabolic health; meanwhile, elevated TC and LDL-C further suggest that lipid homeostasis is already disturbed before overt diabetes develops.

Against this background of systemic lipid metabolic abnormalities, fatty liver may be regarded as a further manifestation of ectopic lipid deposition in the liver and was therefore another important variable strongly associated with prediabetes in the present study. As an important hepatic manifestation of systemic metabolic dysfunction, particularly insulin resistance, hepatic lipid accumulation not only reflects abnormal lipid storage but may also aggravate dysregulation of glucose and lipid metabolism by disrupting hepatic glucose metabolism, increasing hepatic glucose production, and impairing insulin action (25–27). Previous studies have shown that hepatic steatosis is common in individuals with prediabetes, and that some of these individuals may already have an increased risk of liver fibrosis (28). Hepatic steatosis and certain fibrosis-related indices have been associated with an increased risk of future diabetes development (29). Taken together, the present findings suggest that the coexistence of elevated BMI and fatty liver may reflect a metabolic axis involving adiposity, hepatic lipid accumulation, and insulin resistance that is closely related to prediabetes.

Accumulating evidence suggests that abnormalities in glucose and lipid metabolism are not isolated phenomena, but rather part of an interconnected metabolic crosstalk network involving multiple organs, including the liver, adipose tissue, and skeletal muscle (30). Ectopic lipid deposition, together with the abnormal accumulation of bioactive lipids in non-adipose tissues, can directly impair insulin signaling and promote systemic insulin resistance (31). At the same time, adipose tissue inflammation, dysregulated lipolysis, and fatty acid remodeling may increase the flux of free fatty acids and inflammatory signals to the liver, thereby further aggravating hepatic lipid deposition and insulin resistance (32, 33). The liver, adipose tissue, and skeletal muscle can also communicate through long-range regulation mediated by hepatokines, adipokines, and related signaling pathways (34, 35). Furthermore, hepatic insulin resistance may attenuate insulin-mediated suppression of gluconeogenesis while relatively preserving lipogenic pathways, ultimately creating a vicious cycle in which abnormalities in glucose and lipid metabolism coexist and mutually amplify each other (36). This interconnected pattern is consistent with the metabolic characteristics observed in the present study.

Age and hypertension also showed stable associations with prediabetes. Older age was associated with a higher likelihood of prediabetes, which is consistent with the progressive decline in glucose metabolic function with aging. This age-related increase in dysglycemia may be partly attributable to reduced compensatory capacity of pancreatic β-cells and decreased insulin sensitivity. Recent evidence further suggests that recovery of β-cell function is a key determinant of reversion from prediabetes to normoglycemia, underscoring the importance of preserved β-cell function in favorable glycemic trajectories (37). Hypertension was likewise positively associated with prediabetes, supporting the notion that abnormal glucose metabolism, adiposity, dyslipidemia, and blood pressure abnormalities tend to cluster within a system characterized by insulin resistance, chronic inflammation, endothelial dysfunction, and progressive target-organ injury (38, 39).

The positive association of ALT was consistent with its relation to hepatic steatosis and insulin resistance, whereas elevated BUN may reflect increased metabolic burden. The inverse association between TBIL and abnormal glucose metabolism was broadly consistent with previous studies, which have reported that higher TBIL levels are associated with lower risk of type 2 diabetes, lower insulin resistance, and lower inflammatory burden, suggesting a potential role of bilirubin in glucose metabolic homeostasis (40, 41). By contrast, the associations of AST and Scr may have been influenced by body composition, such as muscle mass, and nutritional status, and therefore may not directly reflect underlying pathophysiological mechanisms. Given the complexity of these relationships, the underlying molecular mechanisms warrant further investigation.

This study has several strengths, including its large sample size, the use of standardized health examination data, and the comprehensive evaluation of multiple metabolic indicators. Moreover, by integrating conventional regression with machine learning, the study provided complementary evidence regarding variable importance and potential nonlinear associations. Despite these strengths, several limitations should be considered. Because this was a single-center cross-sectional study, causal inferences cannot be made and the generalizability of the findings may be limited. The participants were recruited from a health examination population in China and primarily comprised Chinese adults, which may limit the generalizability of the findings to other countries, races, and ethnicities. Recent research (42) has shown that racial differences may affect diabetes risk prediction even among individuals with a normal BMI, suggesting that population-specific factors may influence the assessment of glucose metabolism risk. Owing to the relatively limited diversity of the study population, this study was unable to further examine whether the associations between metabolic indicators and prediabetes differ across racial or ethnic backgrounds. Future large-scale, multicenter studies involving populations from different regions of China and diverse international cohorts are warranted to validate and extend these findings. As the study was based on routinely collected health examination data, it could not capture deeper molecular-level metabolic alterations; moreover, residual confounding due to unmeasured variables and potential bias related to single measurements cannot be completely excluded. Also, oral glucose tolerance test data were unavailable, which may have led to under-recognition of isolated impaired glucose tolerance; SHAP analysis improved model interpretability, it reflected model-level contributions rather than direct biological mechanisms. Therefore, the present findings should be interpreted with appropriate caution and further validated in prospective, multi-center studies.

## 5 Conclusion

In summary, in this health examination population, age, hypertension, obesity, fatty liver, and dyslipidemia may be important factors associated with prediabetes, among which elevated BMI and fatty liver showed relatively stronger associations with an increased likelihood of prediabetes.

## Data Availability

The datasets presented in this article are not readily available due to privacy and ethical restrictions. The datasets used and/or analyzed during the current study are available from the corresponding author upon reasonable request. Requests to access the datasets should be directed to the corresponding author.
