# Supplementary material for: Metabolic characteristics and factors associated with prediabetes in Chinese adults based on real-world health examination data: a cross-sectional study
Source: Front Nutr. 2026 Jun 12;13:1850839. doi: 10.3389/fnut.2026.1850839 (PMC13303982; doi:10.3389/fnut.2026.1850839)

Supplementary Material

[Supplementary Table S1 STROBE Statement checklist 2](#_Toc230732032)

[Supplementary Table S2 Variable Coding Scheme for Logistic Regression Analysis 5](#_Toc230732033)

[Supplementary Table S3 Univariate Logistic Regression Analysis 6](#_Toc230732034)

[Supplementary Table S4 Multivariable Logistic Regression Analysis 7](#_Toc230732035)

[Supplementary Figure 1 Confusion Matrix 8](#_Toc230732036)

[Supplementary Figure 2 Marginal SHAP(BMI) contribution and |SHAP| shares of major metabolism-related variables within BMI 29.4–32 kg/m² 9](#_Toc230732037)

**Supplementary Table S1 STROBE Statement checklist**

STROBE Statement—Checklist of items that should be included in reports of ***cross-sectional studies***

|  | Item No | Recommendation | section |
| --- | --- | --- | --- |
| **Title and abstract** | 1 | (*a*) Indicate the study’s design with a commonly used term in the title or the abstract | Title |
|  |  | (*b*) Provide in the abstract an informative and balanced summary of what was done and what was found | Abstract |
| Introduction | | |  |
| Background/rationale | 2 | Explain the scientific background and rationale for the investigation being reported | 1 Introduction |
| Objectives | 3 | State specific objectives, including any prespecified hypotheses | 1 Introduction |
| Methods | | |  |
| Study design | 4 | Present key elements of study design early in the paper | 2.1 Study design |
| Setting | 5 | Describe the setting, locations, and relevant dates, including periods of recruitment, exposure, follow-up, and data collection | 2.2 Data sources and measurements |
| Participants | 6 | (*a*) Give the eligibility criteria, and the sources and methods of selection of participants | 2.3 Participants |
| Variables | 7 | Clearly define all outcomes, exposures, predictors, potential confounders, and effect modifiers. Give diagnostic criteria, if applicable | 2.2 Data sources and measurements |
| Data sources/ measurement | 8* | For each variable of interest, give sources of data and details of methods of assessment (measurement). Describe comparability of assessment methods if there is more than one group | 2.2 Data sources and measurements |
| Bias | 9 | Describe any efforts to address potential sources of bias | 2.2 Data sources and measurements |
| Study size | 10 | Explain how the study size was arrived at | 2.4 Sample size |
| Quantitative variables | 11 | Explain how quantitative variables were handled in the analyses. If applicable, describe which groupings were chosen and why | 2.5 Statistical methods |
| Statistical methods | 12 | (*a*) Describe all statistical methods, including those used to control for confounding | 2.5 Statistical methods |
|  |  | (*b*) Describe any methods used to examine subgroups and interactions | 2.5 Statistical methods |
|  |  | (*c*) Explain how missing data were addressed | 2.5 Statistical methods |
|  |  | (*d*) If applicable, describe analytical methods taking account of sampling strategy | 2.5 Statistical methods |
|  |  | (*e*) Describe any sensitivity analyses | 2.5 Statistical methods |
| Results | | |  |
| Participants | 13* | (a) Report numbers of individuals at each stage of study—eg numbers potentially eligible, examined for eligibility, confirmed eligible, included in the study, completing follow-up, and analysed | 3.1 General Information |
|  |  | (b) Give reasons for non-participation at each stage | 3.1 General Information |
|  |  | (c) Consider use of a flow diagram | 3.1 General Information |
| Descriptive data | 14* | (a) Give characteristics of study participants (eg demographic, clinical, social) and information on exposures and potential confounders | 3.2 Baseline characteristics of participant |
|  |  | (b) Indicate number of participants with missing data for each variable of interest | 3.2 Baseline characteristics of participant |
| Outcome data | 15* | Report numbers of outcome events or summary measures | 3.2 Baseline characteristics of participant |
| Main results | 16 | (*a*) Give unadjusted estimates and, if applicable, confounder-adjusted estimates and their precision (eg, 95% confidence interval). Make clear which confounders were adjusted for and why they were included | 3.3 Logistic regression analysis of factors associated with prediabetes |
|  |  | (*b*) Report category boundaries when continuous variables were categorized | 3.3 Logistic regression analysis of factors associated with prediabetes |
|  |  | (*c*) If relevant, consider translating estimates of relative risk into absolute risk for a meaningful time period | 3.3 Logistic regression analysis of factors associated with prediabetes |
| Other analyses | 17 | Report other analyses done—eg analyses of subgroups and interactions, and sensitivity analyses | 3.4 Machine learning analysis |
| Discussion | | |  |
| Key results | 18 | Summarise key results with reference to study objectives | 4 Discussion |
| Limitations | 19 | Discuss limitations of the study, taking into account sources of potential bias or imprecision. Discuss both direction and magnitude of any potential bias | 4 Discussion |
| Interpretation | 20 | Give a cautious overall interpretation of results considering objectives, limitations, multiplicity of analyses, results from similar studies, and other relevant evidence | 5 Conclusion |
| Generalisability | 21 | Discuss the generalisability (external validity) of the study results | 4 Discussion |
| Other information | | |  |
| Funding | 22 | Give the source of funding and the role of the funders for the present study and, if applicable, for the original study on which the present article is based | Funding |

**Supplementary Table S2 Variable Coding Scheme for Logistic Regression Analysis**

| **Variable** | **Coding** |
| --- | --- |
| Gender | Male=0，Female=1 |
| Smoking history | No=0，Yes=1 |
| Alcohol drinking | No=0，Yes=1 |
| Hypertension | No=0，Yes=1 |
| Fatty liver | No=0，Yes=1 |

**Supplementary Table S3 Univariate Logistic Regression Analysis**

| **Variable** | **β** | **S.E** | **Z** | **P** | **OR (95%CI)** |
| --- | --- | --- | --- | --- | --- |
| **Age** | 0.8296 | 0.0168 | 49.308 | <0.001 | 2.29(2.22-2.37) |
| **Gender** | 0.095 | 0.0289 | 3.287 | 0.001 | 1.10(1.04-1.16) |
| **BMI** | 1.0585 | 0.0177 | 59.854 | <0.001 | 2.88(2.78-2.98) |
| **Smoking history** | 0.0402 | 0.0312 | 1.289 | 0.197 | 1.04(0.98-1.11) |
| **Alcohol drinking** | 0.0393 | 0.029 | 1.355 | 0.176 | 1.04(0.98-1.10) |
| **Hypertension** | 0.8459 | 0.0291 | 29.05 | <0.001 | 2.33(2.20-2.47) |
| **Fatty Liver** | 1.218 | 0.0339 | 35.933 | <0.001 | 3.38(3.16-3.61) |
| **TBIL** | -0.1772 | 0.015 | -11.783 | <0.001 | 0.84(0.81-0.86) |
| **ALT** | 0.2745 | 0.016 | 17.127 | <0.001 | 1.32(1.28-1.36) |
| **AST** | 0.2828 | 0.0183 | 15.412 | <0.001 | 1.33(1.28-1.38) |
| **BUN** | 0.3347 | 0.0157 | 21.256 | <0.001 | 1.40(1.36-1.44) |
| **Scr** | 0.1179 | 0.0165 | 7.133 | <0.001 | 1.13(1.09-1.16) |
| **UA** | 0.2096 | 0.0144 | 14.6 | <0.001 | 1.23(1.20-1.27) |
| **TC** | 0.2984 | 0.0146 | 20.378 | <0.001 | 1.35(1.31-1.39) |
| **TG** | 0.3827 | 0.0187 | 20.419 | <0.001 | 1.47(1.41-1.52) |
| **HDL-C** | -0.175 | 0.0146 | -12.008 | <0.001 | 0.84(0.82-0.86) |
| **LDL-C** | 0.344 | 0.0148 | 23.315 | <0.001 | 1.41(1.37-1.45) |

**Supplementary Table S4 Multivariable Logistic Regression Analysis**

| **Variable** | **β** | **S.E** | **Z** | **P** | **OR (95%CI)** |
| --- | --- | --- | --- | --- | --- |
| **Base model** |  |  |  |  |  |
| **Age** | 0.86344 | 0.01862 | 46.365 | <0.001 | 2.37 (2.29, 2.46) |
| **Gender** | -0.18888 | 0.04401 | -4.292 | <0.001 | 0.83 (0.76, 0.90) |
| **Hypertension** | 0.73212 | 0.03195 | 22.911 | <0.001 | 2.08 (1.95, 2.21) |
| **TBIL** | -0.28738 | 0.01758 | -16.351 | <0.001 | 0.75 (0.72, 0.78) |
| **ALT** | 0.37538 | 0.02829 | 13.269 | <0.001 | 1.46 (1.38, 1.54) |
| **AST** | -0.13575 | 0.0292 | -4.649 | <0.001 | 0.87 (0.82, 0.92) |
| **BUN** | 0.14494 | 0.01891 | 7.664 | <0.001 | 1.16 (1.11, 1.20) |
| **Scr** | -0.107 | 0.02086 | -5.129 | <0.001 | 0.90 (0.86, 0.94) |
| **UA** | 0.20704 | 0.02033 | 10.185 | <0.001 | 1.23 (1.18, 1.28) |
| **Expanded model** | |  |  |  |  |
| **BMI** | 0.9232 | 0.01939 | 47.614 | <0.001 | 2.52 (2.42, 2.61) |
| **Fatty liver** | 1.03032 | 0.03754 | 27.449 | <0.001 | 2.80 (2.60, 3.02) |
| **TC** | 0.08067 | 0.01649 | 4.892 | <0.001 | 1.08 (1.05, 1.12) |
| **TG** | 0.08159 | 0.01783 | 4.575 | <0.001 | 1.09 (1.05, 1.12) |
| **HDL-C** | -0.17011 | 0.01828 | -9.304 | <0.001 | 0.84 (0.81, 0.87) |
| **LDL-C** | 0.15656 | 0.01664 | 9.409 | <0.001 | 1.17 (1.13, 1.21) |

**Supplementary Figure 1 Confusion Matrix**

**
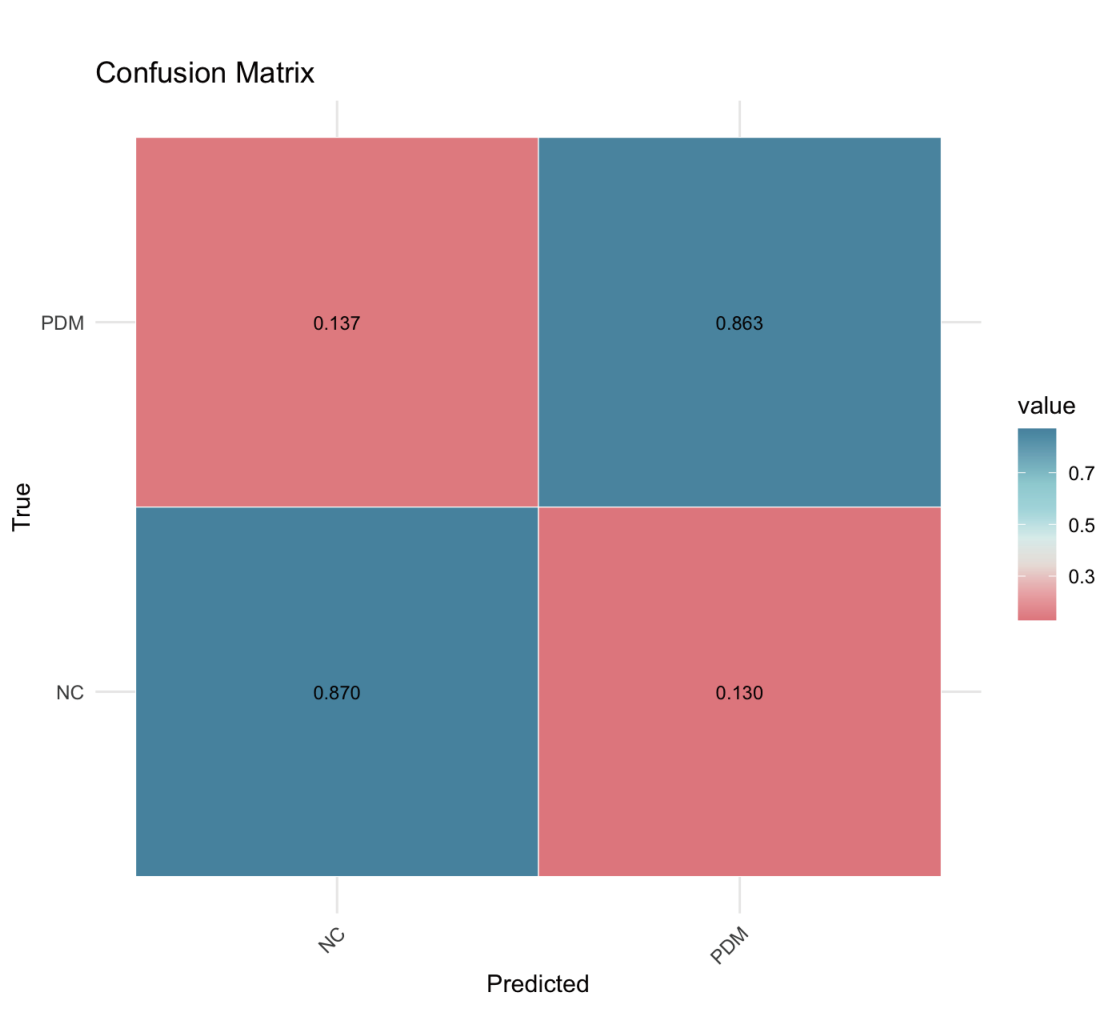
**

**Supplementary Figure 2 Marginal SHAP(BMI) contribution and |SHAP| shares of major metabolism-related variables within BMI 29.4–32 kg/m²**


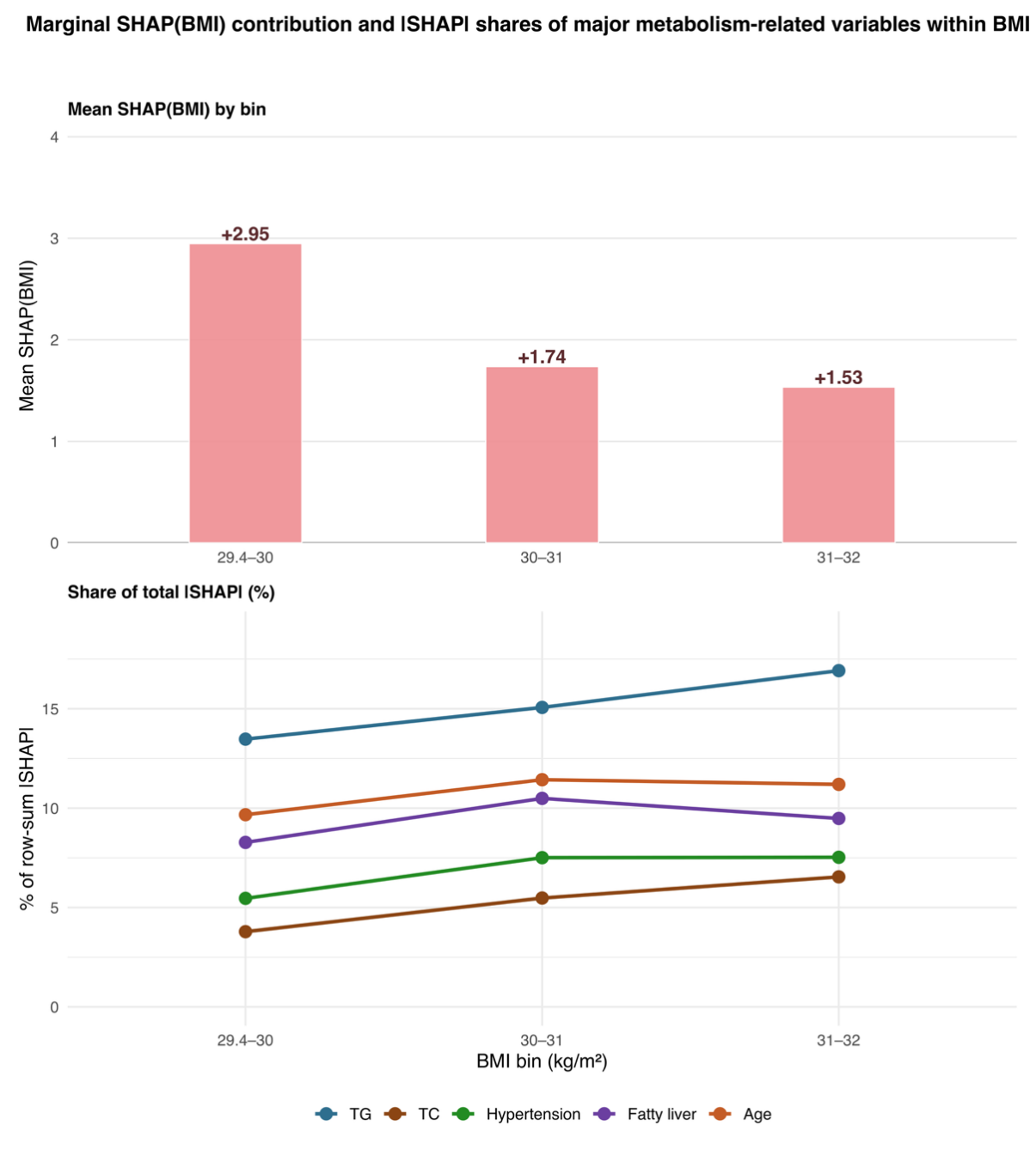

Supplement: Supplementary file 1 [file Supplementary_file_1.docx]
